# A Systematic Review of the Microbiome in Children With Neurodevelopmental Disorders

**DOI:** 10.3389/fneur.2019.00727

**Published:** 2019-07-30

**Authors:** Eleonora Lacorte, Giuseppe Gervasi, Ilaria Bacigalupo, Nicola Vanacore, Umberto Raucci, Pasquale Parisi

**Affiliations:** ^1^National Centre for Disease Prevention and Health Promotion, National Institute of Health, Rome, Italy; ^2^Department of Biomedicine and Prevention, Hygiene and Preventive Medicine School, University of Rome Tor Vergata, Rome, Italy; ^3^Pediatric Emergency Department, Bambino Gesù Children's Hospital, IRCCS, Rome, Italy; ^4^Department of Neurosciences, Mental Health, and Sensory Organs (NESMOS), Faculty of Medicine and Psychology, Sant'Andrea Hospital, Sapienza University, Rome, Italy

**Keywords:** microbiome, neurodevelopmental diseases, gut brain axis, 16S rRNA gene, systematic review

## Abstract

**Background and Purpose:** A relationship between gut microbiome and central nervous system (CNS), have been suggested. The human microbiome may have an influence on brain's development, thus implying that dysbiosis may contribute in the etiology and progression of some neurological/neuropsychiatric disorders. The objective of this systematic review was to identify evidence on the characterization and potential distinctive traits of the microbiome of children with neurodevelopmental disorders, as compared to healthy children.

**Methods:** The review was performed following the methodology described in the Cochrane handbook for systematic reviews, and was reported based on the PRISMA statement for reporting systematic reviews and meta-analyses. All literature published up to April 2019 was retrieved searching the databases PubMed, ISI Web of Science and the Cochrane Database of Systematic Reviews. Only observational studies, published in English and reporting data on the characterization of the microbiome in humans aged 0–18 years with a neurodevelopmental disorder were included. Neurodevelopmental disorders were categorized according to the definition included in the Diagnostic and Statistical Manual of Mental Disorders, version 5 (DSM−5).

**Results:** Bibliographic searches yielded 9,237 records. One study was identified through other data sources. A total of 16 studies were selected based on their relevance and pertinence to the topic of the review, and were then applied the predefined inclusion and exclusion criteria. A total of 10 case-control studies met the inclusion criteria, and were thus included in the qualitative analysis and applied the NOS score. Two studies reported data on the gut microbiome of children with ADHD, while 8 reported data on either the gut (*n* = 6) or the oral microbiome (*n* = 2) of children with ASD.

**Conclusions:** All the 10 studies included in this review showed a high heterogeneity in terms of sample size, gender, clinical issues, and type of controls. This high heterogeneity, along with the small sample size of the included studies, strongly limited the external validity of results. The quality assessment performed using the NOS score showed an overall low to moderate methodological quality of the included studies. To better clarify the potential role of microbiome in patients with neurodevelopmental disorders, further high-quality observational (specifically cohort) studies are needed.

## Introduction

Microorganisms are involved in several different biological mechanisms within the human body, and some of them are crucial for our survival (1, 2). Specifically, the human gut contains more than 100 million bacteria, up to 10–100 times the number of human cells, that have reached, after years of common development, a mutually beneficial symbiotic state with the human body. These cells include six major phyla (i.e., *Firmicutes, Bacteroidetes, Proteobacteria, Actinomycetes, Verrucomicrobia*, and *Fusobacteria*), with *Bacteroidetes* and *Firmicutes* being the dominant ones (3). These several trillions of commensal microbes living in the human gut are collectively referred to as the gut microbiome (4).

Gut microbiome is essential to human health, as it plays a major role in several relevant biological functions. A growing number of reviews reports on the relationship between gut microbiome and the central nervous system (CNS), suggesting that the gut microbiome may have an impact on CNS functions through specific pathways, called microbiota-gut-brain-axis (MGBA) (3–5). The microbiome undergoes a deep developmental process throughout the lifespan, and establishes its symbiotic relationship with the host early in life.

Therefore, early life alteration of this microbiome were suggested as having a potential impact on neurodevelopment, and to have a role in potentially leading to adverse neurodevelopmental and mental health outcomes (5).

The gut-brain axis, in fact, seems to have a role in brain development, as it appears to be involved in influencing microglial maturation and function (3). The immune system seems also to have a role in regulating these interactions. Moreover, the microbiome seems to be involved in regulating mucosal and systemic immune responses, and to have a role in the onset of inflammatory disorders of the CNS due to its involvement in modulating immune responses and antigen mimicry (6–8). This role of the microbiome might also be due to its influence in activating peripheral immune cells, that take part in modulating the responses to neuro-inflammation, brain injury, autoimmunity, and neurogenesis. Thus, the microbiome might be involved in both modulating the production of neurotransmitters, and in synthesizing them *de novo*. Moreover, serotonin is primarily found in the gastrointestinal tract, which suggests that the microbiome might have a role in modulating its production. Similarly, microbiota-dependent pathways specific for the regulation of neurotransmitters have also been hypothesized for GABA, norepinephrine, dopamine, and tryptamine (6).

Several other neurological conditions involving dysimmune mechanisms, such as multiple sclerosis (MS) (7), Pediatric Acute-Onset Neuropsychiatric Syndrome (PAS), and Pediatric Autoimmune Neuropsychiatric Disorders Associated With Streptococcal Infections (PANDAS) (8, 9), have also been associated with imbalances of the gut microbiome composition, thus raising the hypotesis of an involvement of the immune response modulation carried out by the microbiome in their etiological model. These disorders, however, are not included within the definition of neurodevelopmental disorders (NDD), as they can be considered as secondary to infectious, inflammatory, and/or immune mechanisms.

Recent studies also suggested that the human microbiome ecosystem may have an influence on brain's development, central signaling systems, and behavior, thus indicating that gut dysbiosis may be associated with some neurological and neurodevelopmental disorders (2, 10, 11). Some relatively recent studies on animal models report an association between the microbiome and some neuropsychiatric conditions, such as Parkinson's disease (12), and autism spectrum disorders (ASD) (13, 14). Some recent translational studies also showed some efficacy of fecal transplantation in children with autism disorders spectrum (15).

However, an appropriate assessment of the existence and mechanisms of a mutual influence between the microbiome and neurodevelopmental disorders in children would require addressing some major issue. The main aspect is that of all the physiological changes in the composition of the microbiome following the developmental phases should be assessed when attempting to characterize it. Moreover, an adequate assessment of the prevalence of neurodevelopmental disorders, according to both gender and the different developmental stages at different ages should be carried out. Literature on the prevalence of NDDs and mental disorders in children and adolescents has grown significantly over the last three decades worldwide. A relatively recent meta-analysis estimated a 13.4% worldwide-pooled prevalence of any mental disorder, thus making them and their negative consequences a major health priority (16). As for the prevalence of specific disorders, one study reports a 3–5% worldwide prevalence of ADHD in children (17), with gender ratios (F:M) ranging from 1:3 to 1:16, according to country (18). Another study reports a 1% worldwide population prevalence of ASD (19), with a 4–8 times higher prevalence in males compared to females (20). However, data from the US Centers for Disease Control and Prevention (CDC) indicate that as many as 1/80 children have an ASD, and diagnoses have dramatically increased over the last few years (11).

Studying the alpha and beta diversity of the microbiome (21) of children with specific NDDs, could lead to adapting strategies for the management of specific symptoms in these subjects. In particular, the quantification and evenness of microbial composition in single individuals (i.e., alpha diversity), and the differences in microbial composition among individuals (i.e., beta diversity) could improve the knowledge on possible pathophysiological traits of these NDDs, thus leading to potential adaptations in the therapeutic approaches (21).

To our knowledge, only one recently published systematic review focuses on the relationship between gut microbiome and children's physical and mental health. This review found 11 randomized clinical trials on this issue, and concluded highlighting the need for further studies to increase our knowledge on the characteristics of the microbiome in relation to mental health outcomes in children (22). No further systematic reviews on population-based studies in children are available on this topic.

The objective of this systematic review was to identify all available evidence from observational studies specifically aimed at describing the characteristics of the microbiome in children with neurodevelopmental disorders, and investigating potential specific and/or distinctive traits of such microbiome when compared to healthy children.

## Methods

The review was performed following the methodology described in the Cochrane handbook for systematic reviews (23), and was reported based on the PRISMA statement for reporting systematic reviews and meta-analyses (24). All literature published up to April, 2019 was retrieved searching the databases PubMed, ISI Web of Science and the Cochrane Database of Systematic Reviews using the following terms: (microbiom^*^ OR micro-biom^*^ OR microbiot^*^ OR micro-biot^*^ OR “gut brain axis” OR “gut-brain axis” OR gastrointestin^*^ OR gastro-intestin^*^) AND (pediatric^*^ OR pediatric^*^ OR child OR children OR childhood OR infant^*^) AND (CNS OR CNS OR cognit^*^ OR brain OR neurodevelopment^*^ OR neuro-development^*^ OR neuropsych^*^ OR neuro-psych^*^). No restrictions were applied for date of publication, study design, nor language. References of considered studies were also searched to identify any further relevant data. Studies were initially selected by two independent reviewers (NV, EL) based of their pertinence with and relevance to the topic of the review. Disagreements were resolved by discussion between the reviewers.

The full texts of selected studies were retrieved and assessed for inclusion based on the following predefined inclusion/exclusion criteria.

Only observational studies, published in English and reporting data on the characterization of the microbiome in humans aged 0–18 years with a neurodevelopmental disorder were included. Randomized clinical trials, quasi-experimental studies, conference proceedings, abstracts, editorials, reviews, systematic reviews, meta-analyses, case-reports or case-series were excluded. Only studies reporting enough information and data to allow for a comprehensive methodological quality assessment and a summary of findings were included, while studies reporting results in a narrative way or data in a non-quantifiable way were excluded. Neurodevelopmental disorders were classified according to the definition included in the Diagnostic and Statistical Manual of Mental Disorders, version 5 (DSM−5).

Included studies were qualitatively assessed by 6 independent reviewers (PP, NV, EL, IB, GG, and UR) using the Newcastle-Ottawa Quality Assessment scale for Cohort and Case-Control Studies (NOS) (25). The NOS tool includes 8 items addressing the appropriateness of 3 major areas: the selection of study sample, the comparability of study groups, and the ascertainment of either the exposure for case control studies or the outcome for cohort studies. Studies can be assigned a maximum of 9 stars, with a maximum of 4 stars for the Selection area, 2 stars for the Comparability area, and 3 stars for either the Outcome or the Exposure area. Further potential sources of bias or other methodological issues were also addressed.

Data were extracted using specifically-designed standardized forms. Disagreements were resolved by discussion between the 6 independent reviewers (PP, NV, EL, IB, GG, and UR).

## Results

Bibliographic searches yielded 9,237 records. One study was identified through other data sources. A total of 16 studies were selected based on their relevance and pertinence to the topic of the review, and were then applied the predefined inclusion and exclusion criteria. Six studies were excluded as they included adult subjects (26), did not report specific data on the microbiome characterization (27–29), or focused on yeasts (30) or inflammatory processes (31).

A total of 10 case-control studies met the inclusion criteria, and were thus included in the qualitative analysis and applied the NOS score. Results were reported separately, according to the specific NDD considered in the study, and the type of microbiome analyzed (either oral or gut microbiome).

**Figure 1:**
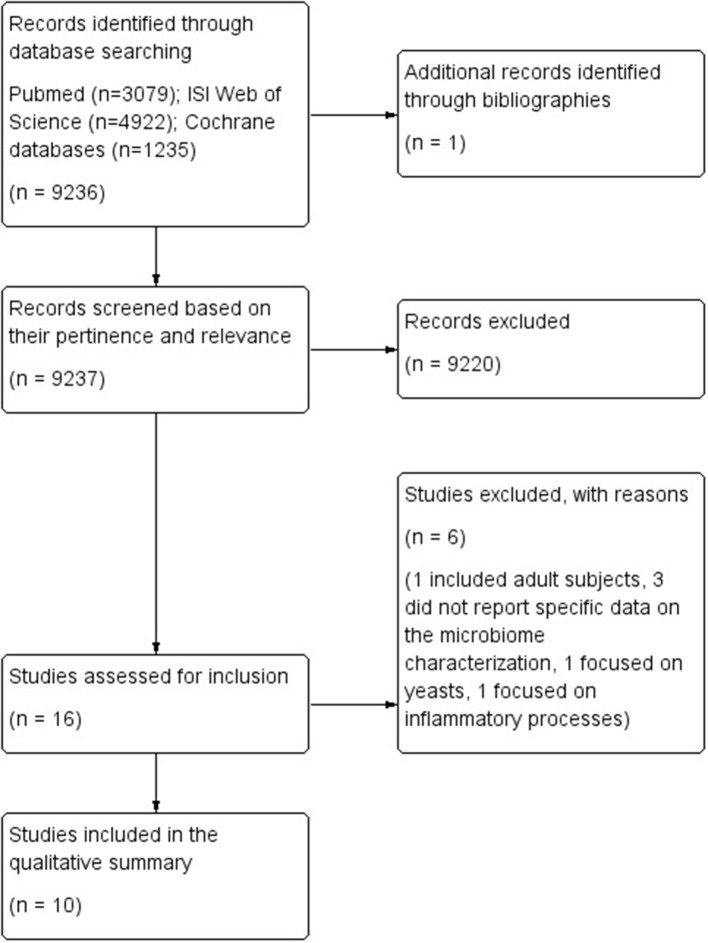
Flow diagram of literature.

The flow diagram of literature is reported in Figure 1.

Two studies reported data on the gut microbiome of children with ADHD compared to children with a neuro-typical (NT) development (32, 33), while 8 studies reported data on either the gut microbiome (*n* = 6) (34–39) or the oral microbiome (*n* = 2) (40, 41) of children with ASD compared with controls with a NT development. All studies were classified as case-control studies, even though all of them analyzed the composition of the microbiome at the time of the sampling, and not in a retrospective way. Table 1 reports a summary of the characteristics and results of each included study.

**Table 1:** Characteristics of included studies.

**Characteristics**							**Quality assessment by the NOS tool**^****§****^			**Summary of findings**		
										**N. of subjects**		**Results**
**First author, year**	**Cases**	**Controls**	**Type of sample**	**Nucleic acids extraction**	**Computational pipelines**	**Potential confounders**	**Selection**	**Comparability**	**Outcome/Exposure**	**n. cases**	**n. controls**	**Data**
**GUT MICROBIOMA**												
Prehn-Kristensen (32)	Males with ADHD diagnosis according to the DSM-IV -TR criteria	Male healthy children recruited through newspaper announcement	Stool samples: collected in fecal collection tubes and stored at 4°C until preparation	Total DNA was extracted using FastDNA^TM^ KIT FOR SOIL	MOTHUR	Ten children with ADHD had been taking medicine for more than 1 year to treat ADHD symptoms. Nine of them discontinued medication for at least 48 h prior to sample collection	*		*	14 (*M* = 14)	17 (*M* = 17)	**Alpha Diversity Index** Chao1: no differences Shannon: ADHD < HC (*p* = 0.036) Observed: no difference. **Correlations with alpha diversity** Hyperactivity: *R* = −0.35 (*p* = 0.03) Impulsivity: non-significant Attention: non-significant
Jiang (33)	ADHD diagnosis according to the DSM-IV criteria	Neuro-typical (NT) control enrolled via advertisements	Stool samples: collected by parents in sterile plastic cups and stored at −20°C at home. Samples were kept in an icebox that was delivered to the laboratory within 30 min, and then stored at −80°C.	Fecal microbial DNA was extracted from 200 mg of feces using the QIAamp DNA Stool Mini Kit	QIIME (version 1.7)	Children who were taking probiotics or antibiotics during the 2 months prior to the fecal sample collection, who had apparent gastrointestinal symptoms, or had been or were currently taking medications for ADHD were excluded from the study sample	**		**	51 (*M* = 38)	32 (*M* = 2)	**OTUs:** 740 in ADHD, 645 in NT **Alpha Diversity Index** ACE: no differences Chao1: no differences Shannon: no differences Simpson: no differences **Correlations with** ***Faecalibacterium*** Total CPDS score: *R* = −0.294 (*p* = 0.037) Hyperactivity: *R* = −0.564 (*p* < 0.001)
Zhai (39)	ASD diagnosis according to DSM- IV-TR and ICD-10 criteria	No reported symptoms of ASD or other neurological disorders	Stool samples: collected by parents and guardians in a small cooler (−4°C) provided by the researchers, and returned on the same day to laboratory where it was homogenized, divided into aliquots and stored at −80°C	Meta-genomic DNA was isolated using a FastDNA Spin Kit for Soil	QIIME software (version 1.9.1)	Subjects were excluded if they were taking mineral supplements or antibiotic medications a month prior to sampling, or if they were in treatment with probiotics and/or prebiotics Controls were excluded if they had stomach/gut problems (e.g. chronic diarrhea, constipation), or if they were related to an individual with autism (e.g., sibling, parent)	*	*	*	78 (*M* = 56)	58 (*M* = 31)	**Alpha diversity:** Chao1 index Increased taxa richness in ASD (*p* = 0.015) **Shannon index::** Significantly higher microbial diversity in ASD (*p* < 0.001) At a genus level, ASD children had a significant increase in *Bacteroides, Parabacteroides, Sutterella, Lachnospira, Bacillus, Biophila, Lactococcus, Lachnobacterium, and Oscillospira* (*p* < 0.01)
Liu (38)	ASD diagnosis according to DSM-5 and ICD-10 criteria	Neuro-typical (NT): typically developing children, without an autism diagnosis and not directly related to an autistic individual	Stool sample: collected and transported to the laboratory for processing within 30 min, where 200 mg samples were preserved in fecal bacteria DNA storage tubes and stored at −80°C	Microbial DNA was extracted from 200 mg fecal samples using the QIAamp Fast DNA Stool Mini Kit	UPARSE	Subjects were excluded if they had a history of use of nutritional supplements or were under special diets None of the included subjects was treated with antibiotics, antifungals, probiotics or prebiotics for at least 3 months before sampling	**	*	*	30 (*M* = 25)	20 (*M* = 16)	**Alpha diversity** SOBS, Chao and ACE indexes showed no significant differences **Beta diversity** Different overall composition At philum level in ASD *Firmicutes* were decreased (*p* < 0.05) while *Acidobacteria* were increased (*p* < 0.05) At family level no difference in *Bacteroidaceae* At taxa level in ASD*Veillonellaceae* and *Enterobacteriaceae* were increased (*p* < 0.05), while *Ruminococcaceae, Streptococcaceae, Peptostreptococcaceae* and *Erysipelotrichaceae* were decreased (*p* < 0.05)
Pulikkan (36)	ASD diagnosis according to DSM-5 criteria using CARS, AIIMS- modified INDT-ASD, and ISAA	Healthy siblings or blood relatives to the ASD children	Stool sample collected from each individual after morning breakfast and were stored at −80°C within 2 h of collection until further processing	DNA from fecal samples extracted using QIAamp Stool Mini Kit	QIIME	All ASD children had normal omnivore native diet similar to healthy subjects and were not on gluten-free diet (GFD) None of the participants took any antibiotic, anti- inflammatory, or antioxidant medication for 1 month prior to the sample collection	**		*	30 (*M* = 28)	24 (*M* = 15)	**Alpha Diversity Index** Shannon: no differences Observed: no differences Phylogenetic diversity: no differences **Beta Diversity** *Principal Component Analysis*PC1 (38.1%): no differences PC2 (11.08%): no differences PC3 (7.43%): ASD (*p* = 0.023)Key families bacteria in differentiating ASD and healthy samples were *Prevotellaceae, Lactobacillaceae, Mogibacillaceae*
Zhang (37)	ASD diagnosis according to the DSM-5 criteria	NT children with no major psychiatric condition according to medical examination and parent interview recruited from 2 kindergartens	Stool samples collected at home by parents and immediately deep frozen, shipped to the laboratory on the same day and stored at −80°C until DNA extraction	Not specified	QIIME's RDP Classifier	None of the included subjects took antibiotics, antipsychotics, probiotics nor prebiotics in the past month prior to sample collection. Children with coeliac disease special diet (such as ketogenic diet) were excluded	***		**	35 (*M* = 28)	6 (*M* = 28)	**Alpha diversity** Shannon index revealed no significant differences between ASD and control group **Beta diversity** Bacterial microbiota of ASD clusters apart from control group (*p* = 0.02) At level of phylum, the ratio*Bacteroidetes/Firmicutes* was higher in ASD (*p* ≤ 0.05), due to an increased relative abundance of *Bacteroidetes* (*p* ≤ 0.05)
Luna (35)	ASD diagnosis based on the Autism Diagnostic Observation Schedule, and a diagnosis of FGID	Neuro-typical (NT) control recruited at the outpatients pediatric GI suite, subdivided according to presence of FGID	Biopsy specimens: placed, immediately after collection, in 2 mL of saline on ice and transported to a laboratory for processing within 15 min, and stored at −80°C	Tissue specimens were processed through MO BIO Power Soil	Modified version of the UPARSE algorithm	None of the participants was taking antibiotics, steroids, nor had any GI infection during the 3 months prior to sample collection	**	*	*	14 (*M* = 14)	Total = 21 (*M* = 18) 15 (NT with FGID) 6 (NT without FGID)	**Beta Diversity (performed by PCA)** *Increased in ASD with FGID Clostridium lituseburense* (*p* = 0.002) *Lachnoclostridium bolteae* (*p* = 0.017) *Lachnoclostridium hathewayi*(*p* = 0.03) *Clostridium aldenense* (*p* = 0.38) *Flavonifractor plautii* (*p =* 0.038) *Terrisporobacter* (*p =* 0.045), after removing a single subjects in NT- FGID*Decreased in ASD with FGID Dorea formicigenerans* (*p* = 0.006) *Blautia luti* (*p* = 0.025)*Sutterella* (*p* = 0.025)
Son (34)	ASD probands from Simons Simplex Collection diagnosed via Autism Diagnostic Observational Schedule, and subdivided according to the presence of FGID	Neuro-typical (NT) siblings recruited via Simons Simplex Collection registry through the Interactive Autism Network, and subdivided according to the presence of FGID	Stool samples: 2 ml of stool collected in a stool “hat” placed in the toilet and immediately transferred into a vial that contained 10 ml of RNA later for metagenomics studies Samples were shipped in cold packs overnight to the laboratory	Fecal DNA was extracted from stool samples immediately upon arrival using ZR Fecal DNA MiniPrep	UCHIME	All included subjects were off probiotics and antibiotics for at least 1 month	**		*	Total = 59 (*M* = 51):25 (ASD with FGID)34 (ASD without FGID)	Total = 37 (*M* = 16) 13 (NT with FGID) 31 (NT without FGID)	**Alpha Diversity Index** Chao1: no differences Shannon: no differences **Beta Diversity** PERMANOVA: No differences Binomial regression analysis: No differences Exploratory analysis: No differences
**ORAL MICROBIOMA**												
Hicks (40)	ASD defined by clinician consensus using the DSM-5 criteria	Children with negative ASD screening based on the Modified Checklist for Autism in Toddlers-Revised, and children who met typical developmental milestones on standardized physician assessment	Saliva samples: collected at the time of enrollment, following an oral water rinse, using an ORAcollect swab from the sublingual and parotid regions of the mouth in a non-fasting state. Swabs were stored at −20°C prior to processing	Salivary RNA was extracted using a standard Trizol technique and the RNeasy mini column	RNA reads	Children with feeding tube dependence, active tooth	*		*	180 (*M* = 153)	106 (*M* = 64) healthy subjects	**Alpha diversity** No difference in Shannon index at both species and phylum level **Beta diversity** Bray-Curtis index showed significant differences between the ASD, TD and DD groups (*p* = 0.04) 12 taxa were different between the ASD, TD and DD groups (FDR < 0.05); 3 taxa were different between the ASD group and the DD group (FDR≤0.05); only *Planctomycetes* differed between ASD and both the TD (FDR = 0.001) and DD group (FDR = 0.02) No differences observed in the *Firmicutes*/*Bacteroides* ratio
Qiao (41)	ASD diagnosis according to the DSM-5 criteria, confirmed with the ICD-10 criteria	Healthy children recruited from primary schools	Saliva samples: 1 ml of non-stimulated, naturally outflowed saliva collected and transferred into 1.5 ml sterile tubes Dental plaques samples: first permanent molars isolated with cotton rolls and gentle air-drying Supra-gingival plaques obtained separately from caries-free molars in 4 quadrants per subject with sterile Gracey curettes, and pooled All samples were immediately placed on ice, transported to the laboratory within 2 h, and stored at −80°C	DNA from dental and salivary samples was extracted with the OMEGA-soil DNA Kit	QIIME (version 1.9.1)	Cases were excluded if in treatment with antibiotics within 3 months before the study, if treated with local antimicrobial agents within 2 weeks, or if they had been using any medication for ASD, sedatives, or were under gluten-free/casein-free (GF/CF) diet or were using probiotics Controls were excluded if they had any systemic or local disorders that caused oral mucosal lesions (e.g. lichen planus), if they had periodontal pockets ≥4 mm, acute oral infection (e.g. abscess), evidence of oral candidiasis, if they were in receiving antibiotics within 3 months before the study, or were treated with local antimicrobial agents within 2 weeks	***	*	*	32 (*M* = 27)	27 (*M* = 21)	**Alpha Diversity Index dental** ACE: ASD < control (*p* < 0.05) Shannon: ASD < control (*p* < 0.05) Shannoneven: ASD < control (*p* < 0.05) **Alpha Diversity Index saliva** ACE: no differences Shannon: no differences Shannoneven: no differences **Beta Diversity Index in ASD** *Streptococcus* and *Haemophilus* (high levels) *Prevotella, Selenomonas, Actinomyces, Porphyromonas*, and *Fusobacterium* (low levels) *Rothia*: high levels in dental, low levels in saliva

§ *Newcastle-Ottawa Quality Assessment scale for Cohort and Case-Control Studies (NOS). Studies can be assigned a maximum of 4 stars in the Selection section, 2 stars in the Comparability section, and 3 stars in either the Outcome or the Exposure section*.

### Qualitative Assessment

All studies were assessed using the NOS tool to evaluate their methodological quality. Two studies (37, 41) were assigned 5 stars, which was the highest score achieved, while 3 studies (33, 35, 38) scored 4 stars, 3 studies (34, 36, 39) scored 3 stars, and 2 studies (32, 40) scored 2 stars, which was the lowest score achieved. The main reasons for such low quality scores was that only one (40) of the included studies specifically stated that the diagnosis in the enrolled cases was confirmed by an independent validation, and none of the included studies used the same diagnostic tool to exclude the diagnosis in the enrolled controls. Moreover, the definition of controls was very heterogeneous across studies, and in some cases, the source of controls was not clearly described. These aspects contributed to introduce the potential for misclassification or inclusion of controls with conditions that might have shared some etiological factors or similar biological and/or behavioral characteristics with cases. Some of the studies enrolled as controls sibling or relatives of cases. Family matching might be useful in some circumstances to match for confounders such as environment and lifestyle habits. However, in this specific case, it might not be adequate, as the considered conditions (ASD in particular) include symptoms specifically affecting lifestyle habits, such as eating behaviors (e.g., food selectivity, refusal, etc.), and psychological symptoms such as anxiety, that might exacerbate GI symptoms. This makes it almost impossible to completely control for dietary habits or GI symptoms and their effects on the microbiome. Variables as dietary habits, use of food supplements and/or medications, GI symptoms, in fact, can strongly affect the microbiome, and should be taken into account when analyzing results from studies investigating the characterization and potential differences in the composition of the microbiome in different populations. This is even more relevant when considering children with ND disorders, that include symptoms specifically affecting lifestyle habits, and have a higher frequency of GI symptoms. Therefore, this complex relationship between ND symptoms and the microbiome also causes the studies to be limited by the potential for reverse-causality bias or simultaneity bias.

Moreover, due to the specific design of all included studies, the NOS item referring to the response rate was interpreted as requiring to report the number of eligible subjects that were actually enrolled in the study and considered in the analyses. Table 2 reports the qualitative assessment of all included studies for each domain of the NOS scale.

**Table 2:** Qualitative assessment of the studies by the Newcastle-Ottawa Quality Assessment scale for Cohort and Case-Control Studies (NOS).

		**Selection**				**Comparability**	**Exposure**			**Total**
	**Diagnosis**	**Is the case definition adequate?**	**Representativeness of the cases**	**Selection of controls**	**Definition of controls**	**Comparability of cases and controls on the basis of the design or analysis**	**Ascertainment of exposure**	**Same method of ascertainment for cases and controls**	**Non-response rate**	**Number of stars**
Prehn-Kristensen (32)	ADHD			*			*			2
Jiang (33)	ADHD			*	*		*		*	4
Zhai (39)	ASD				*	*	*			3
Liu (38)	ASD	*			*	*	*			4
Pulikkan (36)	ASD			*	*		*			3
Zhang (37)	ASD		*	*	*		*		*	5
Luna (35)	ASD	*			*	*	*			4
Son (34)	ASD		*	*			*			3
Hicks (40)	ASD				*		*			2
Qiao (41)	ASD	*	*	*		*	*			5

### Laboratory and Technical Aspects

The most commonly used technique for the identification, classification and quantification of microbiome across included studies was the 16S rRNA gene sequencing. The 16S rRNA gene is a highly preserved component of the transcriptional process of all DNA-based life forms. Therefore, it is highly suitable to be used as a target gene for DNA sequencing in samples containing up to hundreds or thousands of different microbial species.

The preserved regions of the 16S rRNA gene can be targeted using specifically designed universal PCR primers, thus allowing to amplify the gene in a wide variety of microorganisms from a single sample.

Specifically, fecal samples provide wider taxonomic information, targeting the hypervariable regions. Moreover, public databases mostly include sequences corresponding to the hypervariable regions, compared to other regions. Therefore, the partial sequences corresponding to this region will have, within the database, more sequences to be compared with, thus making it easier to perform a phylogenetic analysis.

Species, genera, families, and phyla are conventionally defined with a value of phylogenetic distance of 0.03, 0.05, 0.10, and 0.20, respectively, based on the entire length (almost 1,540 bp) of the 16S rRNA gene sequence (42).

The 16S rRNA gene is sequenced using NGS platforms, and sequences with a generally ≥97% similarity are clustered in Operational Taxonomic Units (OTUs), which are use to discriminate species and classify nucleotide sequences in the different taxonomic levels. The abundance of each OTU is then estimated based on the number of corresponding sequences. The most frequently used measures are the OTU abundance of different species observed in each sample, the phylogenetic diversity, and the Shannon diversity index, that quantifies the diversity of microbial species within a specific community.

### The Findings for Each Study

#### ADHD

##### Gut microbiome

One study (32) enrolled 14 male children with ADHD diagnosed according to the DSM-IV-TR criteria (mean age 11.9 ± 2.5 years), and 17 controls with no neurological diseases (mean age 13.1 ± 1.7 years). All cases and controls were males. All cases were enrolled through a German outpatient department, while controls were recruited via newspaper announcement, and all were reimbursed for participation. Stool samples were collected, DNA was amplified for the variable regions V1 and V2, and sequences were binned in OTUs with 97% similarity. Alpha diversity was calculated using the Shannon diversity and Chao1 indexes, while beta diversity was analyzed using multivariate statistics. To identify potential biomarker OTUs a full linear discriminant analysis (LDA) effect size (LEfSE) was conducted. The study showed significant differences in the microbiome of children with ADHD compared to controls, with ADHD children having a reduced microbial diversity and composition. Children with ADHD showed a higher abundance in the Bacteroidaceae family. At a family level, a higher level of *Prevotellaceae*, Catabacteriaceae, and *Porphiromonadaceae* was found in controls, while higher levels of *Neisseriaceae* were observed in children with ADHD. A significant correlation was observed between levels of hyperactivity and changes in alpha diversity, but no significant correlation was found between the microbiome and clinical symptoms according to the CBCL (Child Behavior Checklist) questionnaire.

The study resulted as having the lowest score in the NOS quality assessment. The main reason for such low score was the absence of a detailed description of the sources from which cases and controls were enrolled, which also affected an adequate assessment of the representativeness of the sample. Another limitation was that no matching or adjustment was adopted in the enrollment phase and/or when analyzing data. A further reason was that the absence of ADHD or any neurological condition in controls was based on parent-reported data, thus meaning that it was not used the same method for ascertaining ADHD in both cases and controls.

The conclusion of this study are, thus, affected by several methodological limitations, as long as limitations reported by the authors themselves, such as the small sample size, and the inclusion among cases of 10 out of 14 children that had been in treatment for more than 1 year with methylphenidate (MPH), a drug that might have had an effect on gut bacteria, and whose impact on microbiome is still unclear.

The second study (33) analyzed the gut microbiome in 51 treatment-naive children with ADHD diagnosed according to the DSM-IV classification (mean age 8.47 ± 8.47 years), and 32 matched neuro-typical (NT) controls (mean age 8.5 ± 8.47 years). A total of 75% of cases and 69% of controls were males. Cases were enrolled from a Chinese child and adolescent outpatient clinical center, while controls were recruited via advertisements. Fecal samples were collected, and the bacterial 16S ribosomal RNA gene V3-V4 region was amplified. High quality sequences were clustered in OTUs picked at a 97% similarity cut-off. Alpha diversity was calculated using the Shannon, Simpson, ACE and Chao1 indexes. Beta diversity was also calculated computing unweighted, weighted UniFrac distances, and a Bray-Curtis Principal Coordinate Analysis (PCoA). Features distinguishing a microbiome specific to ADHD were identified by LDA LEfSe. Analyses showed that *Firmicutes, Bacterioidetes, Proteobateria*, and *Actinobacteria* were the dominant phyla in all samples. No significant differences were observed in these 4 phyla between the ADHD group and the NT group. The ADHD group showed a lower level of Faecalibacterium, Lachnoclostridium, and Dialister. Neither the type of delivery (vaginal vs. Cesarean) nor the type of early feeding (breast vs. formula) had any significant effect on alfa diversity nor on beta diversity. A negative association was observed between Faecalibacterium and both the total CPRS score and the hyperactivity score. A linear discriminant analysis (LDA) effect size (LEfSe) analysis was performed to identify microorganism features that could discriminate fecal microbiome specific to ADHD. At a family level, a significant increase in the relative richness of *Peptostreptococcaceae, Moraxellaceae, Xanthomonadaceae*, and *Peptococcaceae*, and a significant decrease in *Alcaligenaceae* was observed in children with ADHD when compared to controls, while, at a genus level, Faecalibacterium, Dialister, and *Sutterella* were the main phylotypes contributing to the differences between the microbiome composition of children with ADHD and controls.

The study was scored 4 stars in the NOS quality assessment. The main reason for the low score was the absence of a detailed description of the source of both cases and controls, thus affecting both their comparability and representativeness. The study, however, matched cases and controls, and also considered some specific characteristics as potential confounders (e.g., type of delivery and early nutrition). The ascertainment of cases and controls was not performed using the same method, but controls were tested for ADHD using a structured scale (CPRS).

As for the previous paper, the results of this study are limited by some methodological flaws, and by the small sample size. However, authors only included treatment-naive children to avoid the possible effects of drugs on the microbiome, and also excluded children with atopic conditions, as these can be associated to some aspects of the microbiome structure, such as the previously observed association between Faecalibacterium levels and conditions including asthma, eczema, and allergic rhinitis.

#### ASD

##### Gut microbiome

Six studies reported data on the gut microbiome of childen with ASD.

The first study (34) investigated the gut microbiome, and data on functional gastrointestinal disorders (FGID) and diet in 59 children with relatively severe ASD recruited from the Simons Simplex Collection (SSC) sample (43), a resource that includes data from over 2000 families with a single child with ASD and unaffected relatives and siblings gathered from 12 Canadian research clinics (mean age 10.3 ± 1.8 years), and 44 (of which 37 family-matched) NT siblings from the same database (mean age 10.0 ± 1.8 years). A total of 88% of cases and 44% of controls were males. Children were further sub-classified in 4 categories: children with ASD and FGID (*n* = 25), children with ASD and no FGID (*n* = 34), NT children with FGID (*n* = 13), and NT children without FGID (*n* = 31). Stool samples were collected, and bacteria profiles were investigated with broad-range amplification of the V1-V2 and V1-V3 regions of the 16S rRNA gene. Sequences with identical taxonomic assignments were clustered to produce OTUs. Alpha diversity was calculated using the Shannon and Chao1 indexes, while beta diversity was compared in the two groups using a multivariate analysis of variance (PERMANOVA). Due to the potential of over-dispersion in microbiome sequence data, authors used a binomial regression model to investigate the effect of ASD phenotype, FGID phenotype, and interactions between ASD and FGID on each OTU. A targeted qPCR assay was also performed for the *Sutterella, Prevotella* and total *Bacterioidetes* subgroups, and for the C. coccoides-E-rectales group, *Faecalibacterium prausnitzii*, and *Escheirichia coli*. No differences between groups were observed for any of these bacteria. The analysis of alpha diversity showed that ASD, FGID, and ASD+FGID had no significant effect on either OTU complexity or richness. No statistically significant differences were observed in beta diversity between the two groups, nor in the overall microbial composition when analyzed at a phyla level. Both the relative abundances of the *Sutterella* and *Prevotella* genera resulted as having no association with ASD, FGID nor ASD+FGID. Exploratory analyses showed a significant effect of ASD and ASD+FGID on the *Cyanobacteria*/Chloroplast genus, with an increased relative abundance of this genus in children with ASD and FGID. Further inspection of data showed that these results could be due to 2 subjects with higher values of this genus, who had ASD+FGID (constipation) and were consuming chia seeds. When analyzing the V1V2 dataset individually, significant associations were found for ASD and ASD+FGID first order interactions with *Firmicutes*/Asteroplasma, ASD, FGID, and ASD+FGID interactions with *Proteobacteria*/Thalassospira, and FGID and ASD+FGID interactions with *Proteobacteria*/*Burkholderia*. When analyzing the V1V3 dataset, significant associations were observed for ASD and ASD+FGID interactions with *Proteobacteria*/*Comamonadaceae*, ASD, FGID, and ASD+FGID interactions with *Bacterioides*/*Prevotellaceae*, and ASD and ASD+FGID interactions with *Actinobacteria*/*Mobiluncus*.

The study was assigned 3 stars in the quality assessment with the NOS tool. Though authors reported details on the sample recruitment process, a total of 66 families completed the study out of the 245 eligible ones, and no description is provided of the excluded families. Both cases and controls were sampled from a large structured dataset (the SSC). However, the diagnosis of cases was not independently validated. No detailed definition of control was provided, and they were not administered the same diagnostic tool used for cases to exclude a diagnosis of ASD. Moreover, most of the controls (37/44) were family matched, thus introducing the potential for overmatching and the possibility of still having differences in specific variables such as dietary habits.

The second study (35) investigated the mucosa-associated microbial communities in 14 children with ASD and functional gastro intestinal disorders (FGID) (median age 8.5, range 4–13 years), and 21 NT controls both with FGID (*n* = 15, median age 10.5, range 3–18 years) and without FGID (*n* = 6, median age 5.5, range 3–14 years). All children in the ASD/FGID group, and 18 of the NT controls were males. Both cases and controls were recruited from a US outpatient pediatric GI unit among children that were undergoing lower endoscopy for other reasons and had normal colonoscopies. Biopsy samples were collected, and the V1V3 and V4 regions of the 16S ribosomal RNA gene were amplified and sequenced. After quality filtering, OTU clustering was performed. Differences among the 3 groups were tested using principal component analysis (PCA), while an analysis of variance was used for multiple group comparisons. The PCA showed differences between the ASD-FGID group and both the NT-FGID group and the NT group, with no overlap between the NT and the NT-FGID groups. Use of medications resulted as having no effect on the microbiome. Clostridiales, Bacteroidales, Verrucomicrobiales, Burkholderiales, and Enterobacteriales were the most abundant orders across all groups. Age and gender had no effect on the differences among groups. Analyses at an OTU level showed higher levels of Clostridiales, specifically Clostridium lituseburense, Lachnoclostridium bolteae, Lachnoclostridium hathewayi, *Clostridium aldenense*, and Flavonifractor plautii, in the ASD-FGID group, and lower levels of *Dorea formicigenerans* and Blautia luti, along with lower levels of *Sutterella* in the ASD-FGID group. A significant association was also observed between Terrisporobacter species and ASD-FGID. The NT-FGID group also showed higher levels of *Faecalibacterium prausnitzii, Roseburia intestinalis, Oscillospira* valericigenes, and *Bilophila wadsworthia* when compared to NT children. No overlap was found between the organisms associated with specific GIs and those discriminating the ASD group from the NT groups.

The study scored 4 stars in the assessment with the NOS tool. Though cases were required to have a confirmed diagnosis, controls were not screened with the same diagnostic tool. The process of sample selection was reported, along with the reasons for exclusion. However, both cases and controls were recruited among children that were undergoing colonoscopy due to abdominal pain, altered stool patterns, or painless bright red blood per rectum, thus making the sample less generalizable and representative of the reference population.

The third study (36) analyzed and compared the gut microbiome of 30 children with severe ASD (CARS score >36.5) diagnosed using the Childhood Autism Rating Scale (CARS), the DSM-5 approved AIIMS-modified INDT-ASD (INCLEN Diagnostic Tool for Autism Spectrum Disorder), and the Indian Scale for Assessment of Autism (ISAA), recruited from the Sunrise Hospital in Kerala (median age 9.5, range 3–16 years), and 24 family matched (mostly siblings or blood relatives) healthy children (HC) diagnosed as having no ASD nor gastric problems by the family physician (median age 9.5, range 3.5–16 years). A total of 93% of the cases, and 62% of the controls were males. Stool samples were collected, and extracted DNA was amplified targeting the V3 region of the 16S rRNA gene. High-quality reads were clustered in species-level OTUs at a ≥97% identity. A further analysis was carried out by meta-analyzing data from the study with a dataset of 20 children with ASD and 20 controls from the US population downloaded from NCBI. Alpha diversity was calculated using the observed species, Shannon, and phylogenetic diversity indexes, while beta diversity was calculated using UniFrac distances. Genus abundance was also calculated to identify possible discriminating genera using LEfSe and Boruta. A multivariate analysis of variance (PERMANOVA) was also carried out to assess the effect of age, BMI, and autism. No significant differences were observed in both alpha diversity and phylogenetic diversity between children with ASD and HCs. Multivariate analysis showed no significant effect of the considered covariates on microbiome profiles, while a specific analysis of families with principal components analysis (PCA) showed a correlation between disease state and PC3 (namely Pearson's correlation coefficient with FDR adjusted *p* < 0.05). Moreover, PCA showed that higher PC3 values were positively correlated with higher levels of *Lactobacillaceae*, Mogibacteraceae, and *Enterococcaceae*, and negatively correlated with higher levels of *Prevotellaceae*. *Prevotellaceae, Lactobacillaceae*, and Mogibacteraceae resulted as being the 3 key families discriminating samples from children with ASD from samples from HCs. *Firmicutes, Bacteroidetes*, and *Proteobateria* were the 3 most abundant phyla in all samples, with an almost equal proportion of *Bacteroidetes* and *Firmicutes* in HCs, and a higher proportion of *Firmicutes* in children with ASD. A higher level of *Prevotellaceae* was observed in HCs, while a significantly higher relative abundance of *Lactobacillaceae, Bifidobacteriaceae*, and Veillonellaceae was observed in children with ASD. At a major general level, a significantly higher relative abundance of *Bifidobacterium*, Lactobacillum, Megasphera, and Mitsuokella was also observed in children with ASD. Results of the meta-analysis of the OTUs from both Indian and US data showed, among genera common in both Indian and US populations, a significantly higher abundance of *Lactobacillus* in children with ASD.

The study scored 3 stars in the qualitative assessment with the NOS tool. The diagnosis of cases was not independently validated, and authors acknowledge that they were not able to use the most recent tools, as these were not currently available in India. Controls were diagnosed as not having ASD by GPs, but with different tools than those used for diagnosing cases. Cases and controls were also family matched (mainly siblings or blood relatives) with the aim of controlling for diet, environment, and other lifestyle habits. However, all children with ASD had gastrointestinal symptoms and a significantly lower BMI, probably due to GI symptoms. Family matching could have also lead to overmatching for some characteristics that may be implied in the etiology of microbiome imbalances. Moreover, as ASD usually include symptoms specifically related to eating habits (e.g., food selectivity, refusal, etc.), it may be actually impossible, even including siblings as controls, to completely control for dietary habits.

Three studies analyzed the microbiome in non-Western diet children either typically developing or with ASD.

One study (37) investigated the microbiome in non-Western children with ASD compared to typically developing children (TD). A total of 40 children with a diagnosis of ASD according to the DSM-5 criteria were enrolled from a Chinese family fraternity group, and 7 TD children were recruited from kindergartens. Stool samples were collected by their parents, and DNA was sequenced. Filtered sequences were classified to obtain OTUs, and alpha and beta diversity were calculated. Due to low quality reads, 5 children with ASD and 1 TD child were excluded from analyses, thus leaving a final sample of 35 children with ASD (mean age 4.9 ± 1.5) and 6 TD children (mean age 4.6 ± 1.1). Children with ASD showed a different bacterial abundance at both a phylum and a genus levels, with a higher *Bacterioidetes*/*Firmicutes* ratio due to a higher relative abundance of *Bacterioidetes*. *Sreptococcus, Veillonella*, and *Escherichia* were significantly lower in children with ASD, while *Bacterioides, Faecalibacterium, Lachnospiraceae*_*unclass*, and *Oscillospira* were abundant in both groups. No significant differences between groups were observed in alpha diversity (Shannon index), while beta diversity showed that children with ASD cluster apart from TD children. The study observed that the different alteration of the *Bacterioidetes/Firmicutes* ratio between Chinese and Western children with ASD might have been due to differences in the environment and dietary habits, thus underlining the relevance of this issue when analyzing the characteristics of the microbiome.

The study scored 5 stars at the quality assessment with the NOS tool. Both cases and controls were community-based and representative of the source population, and the number of enrolled children that were excluded from the analyses due to unusable data was reported. However, results from this study are still limited by the small sample size.

The second study (38) analyzed the microbiome of Chinese children with ASD compared to neuro-typical (NT) controls. A total of 30 children with a diagnosis of ASD according to the DSM-5 criteria and the ICD-10 classification (mean age 4.43 ± 1.47; 25 males) were enrolled from the Fifth and Third Affiliated Hospital of Zhengzhou University, and 20 age- and sex-matched NT health volunteers (mean age 4.28 ± 1.00 years; 16 males) were enrolled, unrelated to the cases. Gastrointestinal (GI) symptoms were assessed, and fecal samples were collected. DNA was extracted from the samples, and the 16S rRNA gene was amplified with primers specific for the V3-V4 hypervariable regions. OTUs were clustered with a 97% cutoff of similarity, and were applied rarefaction to reduce heterogeneity. Alpha and beta diversity were calculated, and LEfSe was computed. Constipation resulted as having a higher frequency in children with ASD, while none of the children had diarrhea nor abdominal pain. No significant differences in total GI symptoms scores were observed between groups. No differences in alpha diversity (SOBS, Chao, ACE) were observed between groups, even though Shannon and Shannoneven indexes suggested less diversity and evenness in children with ASD. Beta diversity showed an overall different composition of the microbiome of children with ASD compared to NT children. Results showed a lower relative abundance of *Firmicutes* and a higher relative abundance of Acidobacteria in children with ASD, at a phylum level. At a family level, Bacteroidaceae was the most abundant family in all children, with no differences in relative abundance between groups. At a taxa level, the ASD group showed a lower abundance of *Ruminococcaceae, Streptococcaeceae, Peptostreptococcaceae*, and *Erysipelotrichaceae*, and a higher abundance of Veillonellaceae and *Enterobacteriaceae*. At genus level, the ASD group had higher abundance of *Megamonas*, while the NT group had a higher abundance of *Eubacterium* and *Lachnospieraceae-NC2004-group*. A specific association was observed between *Fusobacterium, Barnesiella, Coprobacter, Olsenella, Allisonella*, and *Actinomycetaceae* and subjects with ASD and constipation, while subjects with ASD and no constipation had higher levels of Holdemanella compared to subjects with ASD and constipation and NT subjects with no constipation.

The study was assigned 4 stars at the qualitative assessment with the NOS tool. Cases were ascertained by 2 neuropsychiatrits, and controls were age- and sex-matched. However, the source of cases was not specified, and cases and controls were not diagnosed using the same criteria.

The third study (39) characterized the profile of the microbiome of subjects with ASD compared to healthy children. A total of 78 children (mean age 4.90 ± 1.01 years, 56 males) with a diagnosis of ASD according to the DSM-IV-R and ICD-10 criteria were enrolled from the 3 Chinese provinces, and 58 age and region-matched healthy children (mean age 4.90 ± 0.97 years, 31 males) with no report of ASD symptoms nor other neurological disorders. A total of 88 stool samples were collected by parents or guardians, and DNA was extracted. The V3-V4 region of the 16S rRNA was amplified using PCR, and high quality sections with a similarity >97% were clustered in OTUs. The analysis of alpha diversity showed a higher richness in taxa (Chao1 index) and a significantly higher microbial diversity (Shannon index) in the microbiome of children with ASD. *Bacterioides* was the most abundant genus (>30%) in both groups. At a genus level, ASD children had a significant increase in *Bacteroides, Parabacteroides, Sutterella, Lachnospira, Bacillus, Biophila, Lactococcus, Lachnobacterium*, and *Oscillospira* (LDE effect size). However, as over 80% of the children with ASD had GI symtpoms including abdominal pain, constipation, and dyspepsia, such findings could also have been due to the influence of these GI symptoms on gut functions, diet, etc.

The study was assigned 3 stars at the quality assessment with the NOS tool. Cases were diagnosed using standardized criteria, but controls were not applied these same criteria to exclude a diagnosis. Moreover, though cases and controls were matched for age and region, no details are provided on the source (setting) from which they were enrolled.

#### Oral Microbiome

Two studies reported data on the characterization of the oral microbiome in children with ASDs compared to NT children.

The first study (40) investigated the oral microbiome of the oropharynx of 180 children with ASD (mean age 53 ± 16 months, 85% males), 106 typically developing (TD) children (43 ± 16 months, 60% males), and 60 children with non-autistic developmental delay (DD) (mean age 50 ± 13 months, 70% males). Data on GI disorders were also collected, with ASD children resulting as having a higher rate (22%) of GI disorders compared to TD group (3%), but not compared to the DD group (20%), and a higher rate of food or medicine allergies (21%) compared to both the TD (9%) and DD groups (8%). Saliva samples were collected at enrollment from all participants, and RNA was quantified with next generation sequencing. Differential abundance was calculated only for taxa with raw read counts ≥10 in ≥20% of samples, while mapping was limited to transcripts present at raw read counts of ≥5 in ≥10% of samples. Taxa with higher abundance and prevalence were reported at species and phylum level. Alpha (Shannon index) and beta diversity (Curtiss index) were calculated and compared across groups. Differences in taxa were represented through a multivariate partial least square discriminant analysis (PLS-DA). A total of 41 of the 753 taxa meeting the predefined criteria were present in all samples, with the core oral microbiome including 10 taxa (*Streptococcus, Streptococcus pneumoniae, Gemella* sp. *oral taxon 928, Streptococcus mitis, Neisseria, S. mitis, Proteobacteria, Paseurellaceae, Flavobacteriaceae, Streptococcus* sp. *oral taxon 064*). The most abundant phyla in all samples was *Firmicutes*, with *Lactobacillales* and *Bacillales* as the most prominent orders within the *Firmicutes* phylum. No differences in alpha diversity were observed between the 3 groups at both species and phylum levels, while significant differences were observed in beta diversity, with the TD group showing the higher between-sample diversity, and the DD group showing the least distribution compared to the other 2 groups. Two taxa (*Limnohabitans, Plactomycetales*) were higher in ASD group, and 4 (*Ramlibacter tataouinensis, Mucilaginibacter, Bacterioides vulgatus, Gemmata*) were lower compared to the TD group, while 2 taxa (*Brucella, Enterococcus faecalis*) were higher in the ASD group and 1 (*Flavobacterium*) was lower compared to the DD group. No differences were observed in phyla between children with ASD and GI disorders and children with ASD and no GI disorders, while significant differences between groups were observed in 28 taxa, with 3 of these being downregulated and 25 upregulated, and none of them overlapping with the taxa identified in the comparisons with the DD and TD groups.

The study was assigned 2 stars at the quality assessment with the NOS tool. All diagnoses in the cases were based on the DSM-5 criteria, but no description of the source of both cases and controls was provided, thus making it impossible to asses for selection bias. Moreover, controls were not screened for absence of the condition using the same tool as for cases, and potential confounding factors were not accounted for neither during enrollment nor in the analysis of data.

The second study (41) analyzed possible alterations of the oral microbiome of children with ASD compared to healthy controls. A total of 32 children with a diagnosis of ASD according to the DSM-5 criteria were recruited from the Shangai Children's Medical Center (mean age 10.02 ± 1.43), and 27 gender and age matched healthy controls (HC) were enrolled from primary schools (mean age 10.19 ± 0.59). A total of 84% of cases and 78% of controls were males. All enrolled children provided salivary samples, while only 26 children with ASD and 26 healthy controls provided dental samples. DNA from both salivary and dental samples was extracted, and the V3-V4 region of the 16S ribosomal RNA gene was amplified. High quality reads were clustered in OTUs selected at a 97% nucleotide similarity cut-off, that were used to assess alpha diversity indexes (ACE, Shannon, and Shannoneven) and Good's coverage. Beta diversity was calculated using Student's *t*-test and PERMANOVA. Taxonomy data were used in LEfSe. Results in dental samples showed a significantly lower alpha diversity in children with ASD compared to HC, while analyses of the salivary samples showed no differences among groups. The taxonomic analysis showed *Firmicutes, Proteobacteria, Actinobacteria, Bacteroidetes*, and *Fusobacteria* as the predominant phyla in both salivary (98.06% of the microbes) and dental (94.86% of the microbes) samples of both children with ASD and HC, with a higher predominance of *Proteobacteria* in children with ASD. Children with ASD also showed higher abundance of *Streptococcus* and *Haemophilus*, and lower levels of *Prevotella, Selenomonas, Actinomyces, Porphyromonas*, and *Fusobacterium*, while their levels of *Rothia* were higher in dental samples, but lower in salivary samples. Analyses of a network plot of co-occurrence relationships showed that ASD was associated to a lower interconnection between OTUs, that were thus gathered in smaller, less interconnected, clusters.

This study was assigned 5 stars in the NOS assessment, which was the highest quality score achieved. All the diagnoses of the enrolled cases were independently confirmed at a mental health center, and controls were matched with cases for the main confounding variables. However, the definition and ascertainment of controls was not detailed, and controls did not undergo the same diagnostic procedure as cases to exclude a diagnosis of ASD.

## Discussion

Studying the possible influence of microbiome diversity on normal neurodevelopmental processes is a recent matter of debate. The relevant implications that this could have in terms of diagnostic and therapeutic approaches lead several authors to investigate the possible role of alterations of the microbiome in the onset neurodevelopmental disorders in children. However, most of these studies show some methodological issues affecting their results.

All the 10 observational studies included in this review, in fact, showed several crucial limitations. One first aspect was the high heterogeneity in sample size, gender distribution, and in the definition of cases and controls, along with the diagnostic tools adopted for assessing them. The overall sample size of the included studies was small, ranging from 31 (14 ADHD and 17 controls) to 83 (51 ADHD and 32 controls) subjects in studies on ADHD (25, 26), and from 35 (14 ASD 21 controls) to 286 (180 ASD and 106 controls) subjects in studies on ASD (35, 40). Only one study included subjects with equally distributed genders (32), while in the remaining studies the percentage of males was always higher in cases than controls, with values ranging from 75 to 100% of males in cases, and 44 to 86% in controls. The diagnosis of ADHD was based on two different versions of the DSM/IV (32, 33), while children were diagnosed ASD based on either the Autism Diagnostic Observation (ADOS) (34, 35), the DSM-IV-TR criteria, or the DSM-5 criteria, confirmed, in one case, with the ICD-10 criteria (41).

In another systematic review of RCTs by Kan et al. half of the included studies showed a positive effect of all interventions on psychological well-being. However, the risk of bias of all of the 11 included RCTs was high, and studies did not specify how they handled missing data (22). Moreover, all the studies included in the review by Kan et al. had a small sample size, as did all the studies included in the present review. Being both ASD and ADHD not rare nor substantially uncommon, such small sample sizes do not allow for a high confidence in the observed results.

This high heterogeneity determined a high variability in the reported results, and strongly limited their external validity. In fact, the results of the included studies investigating the gut microbiome in children with ASD showed inconsistent data, as one study (34) found no significant differences in diversity and richness between the microbiome of children with ASD when compared with controls (either unrelated healthy controls or neuro-typical siblings). However, as underlined by the authors, even significant differences observed between cases and controls have been reported in literature to be attributable to a normally high variability in the composition of the gut microbiome in different populations, or to be due to different dietary habits, ages, or even caused by the use of some medications or supplements. Similar results are also reported in one of the included studies investigating the gut microbiome of children with ADHD, that reported a negative association between the abundance of *Faecalibacterium* and parental reports of ADHD symptoms. The authors underlined that the observed variability could be due to differences in the enrolled sample, dietary habits, age, and use of medications. In fact, higher levels of Faecalibacterium were also observed in subjects exposed to a long-term Mediterranean diet, that has vegetables, fruits, nuts, legumes, and unprocessed cereals as its main components. As for the impact of age on the microbiome composition, even though physiological changes in the bacterial microbiome after the age of 3 years are very small, the heterogeneity and inconsistency of study results may be partly explained by age differences in the study samples.

Though it would be very useful to identify potential alterations of the microbiome that could be able to allow an early identification and diagnosis of neurodevelopmental disorders, currently available evidence is inconsistent, and does not provide significant evidence on the use of microbiome as a potential marker for the characterization nor the diagnosis of neurodevelopmental disorders.

The utility of microbiome composition as a marker for ASD was actually investigated in 1 of the studies that analyzed the oral microbiome in children with ASD. However, currently available evidence does not provide significant data on the use of microbiome composition as a marker for the characterization nor the diagnosis of ASD.

Similarly, one of the included studies compared the microbiome diversity of ADHD children with the microbiome diversity of their parents and siblings. Results showed that children with ADHD shared slightly more OTUs with their fathers than with their mothers, while controls shared equal amounts of OTUs with both their fathers and mothers. This suggests that an analysis of parents' microbiome might be of some interest in future studies.

The quality assessment performed using the NOS score showed an overall low to moderate methodological quality of all included studies. None of the studies achieved the higher possible score (9 stars), while only 2 studies (37, 41) were assigned 5 stars.

When summarizing results from the included studies, some specific biases emerged, and they had to be taken into due consideration and discussed when interpreting data and drawing conclusions.

First, 9 out of the 10 included studies did not seek an independent validation of the diagnosis in the enrolled cases, and none of the included studies used the same diagnostic tool to both diagnose the condition in cases, and exclude the diagnosis in the enrolled controls. Moreover, the definition of controls was highly heterogeneous across studies. In fact, only 2 studies enrolled children from primary school, while 2 studies enrolled siblings or blood relatives, and the remaining studies included subject recruited via newspaper announcement, advertisements, or pediatric units. Both issues contribute to introduce the potential for misclassification bias. This bias is usually caused by the use of a diagnostic procedure with an inadequate sensitivity and/or specificity to discriminate the considered exposure and/or outcome, thus causing the incorrect classification of some unexposed/non-cases subjects as exposed or having the outcome (cases), and vice-versa (44).

All studies were classified as case-control studies, even though the case-control design was adopted in an untraditional way. Case-control studies are designed to assess exposures that are antecedent to the onset of the disease, and thus can be considered as risk factors, involved in the etiological mechanisms of the disease/condition in study. All of the included studies, instead, enrolled cases and controls and analyzed the composition of the microbiome at the time of the sampling, and not in a retrospective way. The objective of these studies was, as in case-control studies, to analyze a possible association between the microbiome or the presence of specific bacteria, and the onset or severity of the disease. However, the biological analyses of the microbiome, as performed in these studies, meaning at the time of the enrollment, prevented the studies from investigating the microbiome specifically as a possible risk factor for each considered condition, allowing them to only provide a description and characterization of the microbiome and its possible differences between diseased and non-diseased subjects. Therefore, it is very difficult to discriminate between, or to draw conclusions, on the possible characteristics of the microbiome that could be included in an etiological model of the disease, as their onset is prior to the onset of the disease and thus are them potential causes or part of the causes of the disease, and those that are, instead, subsequent to the onset of the disease, and therefore could be caused by the disease itself or by its specific symptoms (e.g., selectivity in food choice, bad eating habits, stress). All included studies are, therefore, limited by either “reverse causality bias” or “simultaneity bias.” Reverse causality bias is due to considering the investigated exposure as the cause or risk factor for the observed outcome, while it is instead the outcome that is causing the exposure. Simultaneity, instead, occurs when the investigated exposure is at the same time cause and caused by the investigated outcome. Thus, ND symptoms, in this case, could cause changes in behaviors (e.g., dietary habits, food selectivity, etc.) leading to unbalances in the microbiome, which could in turn exacerbate symptoms by causing further discomfort and stress and lead again to even more severe changes the behaviors.

None of the studies calculated a sample size based on a predefined hypothesis. This prevented them from defining an a priori probability of being wrong on false negatives and false positives. We understand that all of these studies can be considered as explorative, and that, in this phase, the absence of evidence on the association between microbiome and presence/absence of a condition does not mean an evidence of a negative association between microbiome and presence/absence of the condition. However, we believe that further, high-quality studies are needed to better understand this topic, including studies on oral, gut and urinary microbiome. In particular, the microbiome should be characterized at birth, and should subsequently be reassessed at regular intervals until adulthood. The possible presence of subjects with a diagnosis of neurodevelopmental disorders should then be assessed. In fact, we deem high-quality cohort studies might be the best-fitted study design to assess the effect of microbiome diversity on neurodevelopment in children. Moreover, as emerged from the selected articles, metagenomic analysis approaches seem to be the most promising and rapidly advancing techniques in this field. However, these techniques should be standardized and accurately reported in publications, to allow for transparency, reproducibility, and comparability of results.

To our knowledge, only one study is available with a design close to the ideal one.

Carlson et al. investigated whether the composition of the microbiome at 1 year of age was associated with cognitive outcomes. They recruited 89 typically-developing 1 year old infants (either twins or singletons) from 2 prospective longitudinal studies of early brain development at the University of North Carolina. Cognitive outcomes were assessed using the Mullen Scales of Early Learning, and global and regional brain volumes were evaluated with structural MRI at 1 and 2 years of age. Fecal samples were also collected from all enrolled children. Cluster analyses identified the presence of 3 groups of infants, defined by their gut bacterial composition. Significant differences between clusters were observed at 2 years in Mullen scores (45).

In conclusion, to better clarify the potential role of microbiome in patients with neurodevelopmental disorders, further accurate and reliable evidence is needed, that can be provided only by carrying out high-quality observational, and specifically cohort studies. In fact, considering the growing interest in this topic, and also the high financial, market investments in this field (i.e., dietary and nutritional supplements), a growing number of studies will be published that will be close to impossible to transfer to routine clinical practice if not carried out with an appropriate design and methodological rigor, which is required and crucial for these studies to be of benefit to patients and their families or caregivers.

## Data Availability

All datasets for this study are included in the manuscript and/or the supplementary files.

## Author Contributions

PP, UR, NV, and EL contributed conception and design of the study. GG, IB, and EL organized the database. EL wrote the first draft of the manuscript. PP, UR, NV, IB, and GG wrote sections of the manuscript. All authors contributed to manuscript revision, read, and approved the submitted version.

### Conflict of Interest Statement

The authors declare that the research was conducted in the absence of any commercial or financial relationships that could be construed as a potential conflict of interest.
